# AGE DIFFERENCES IN THE CHARACTERISTICS AND HABITS OF 2019 BIKE WEEK ATTENDEES IN DAYTONA BEACH, FLORIDA

**DOI:** 10.1093/geroni/igz038.3532

**Published:** 2019-11-08

**Authors:** Felicia V Wheaton, Desmond Brown

**Affiliations:** Bethune-Cookman University, Daytona Beach, Florida, United States

## Abstract

Older adults are an important demographic group in the tourism sector, particularly in Florida. This study assessed age-group differences in the characteristics and habits of 2019 Daytona Bike Week attendees, as evidence indicates the average age of bike week visitors is rising. Aging Studies and Hospitality undergraduate students at Bethune-Cookman University interviewed 335 participants using Google Forms on their mobile phones. Results from chi-square tests indicate that there were significant differences between younger (20-39 years), middle-aged (40-59 years) and older adults (60+ years) in terms of gender and income, expenditures on accommodations, type of accommodations, nights spent, and first-time attendance. A greater percentage of older bike week attendees were male and higher income, and were less likely to be first-time attendees. Older adults also tended to spend more on accommodation and stayed longer, and were more likely to stay in Air BnBs, rental homes or a second home/condo compared with younger and middle-aged adults, but were less likely to stay in a hotel/motel or with friends/family. No significant differences were found in expenditures on food, alcohol, gas, entertainment or shopping. Findings suggest that older bike week attendees are important contributors to bike week tourism, particularly with respect to accommodations.

